# Solitary plasmacytoma of the skull compressing the superior sagittal sinus: Case report and literature review

**DOI:** 10.1016/j.bas.2025.105857

**Published:** 2025-10-30

**Authors:** Biyan Nathanael Harapan, Veit M. Stöcklein, Hanna Zimmermann, Viktoria Ruf, Jochen Herms, Florian Ringel, Michael Schmutzer-Sondergeld

**Affiliations:** a Department of Neurosurgery, LMU University Hospital, LMU Munich, Marchioninistraße 15, 81377, Munich, Germany; b Department for Diagnostic and Interventional Neuroradiology, LMU University Hospital, LMU Munich, Marchioninistraße 15, 81377, Munich, Germany; c Center for Neuropathology and Prion Research, Faculty of Medicine, LMU Munich, Feodor-Lynen-Straße 23, 81377, Munich, Germany

**Keywords:** Intracranial plasmacytoma, Skull plasmacytoma, Solitary plasmacytoma, Plasmacytoma, Superior sagittal sinus

## Abstract

**Introduction:**

Solitary plasmacytomas are rare, localized plasma cell neoplasms without systemic involvement. Intracranial manifestations, particularly those compressing the superior sagittal sinus, are exceptionally uncommon and can mimic more common dural-based lesions such as meningiomas.

**Research question:**

This study aims to highlight the diagnostic and therapeutic considerations in cases of intracranial solitary plasmacytoma involving superior sagittal sinus compression through a clinical case and a targeted literature review.

**Material and methods:**

We report a case of a 52-year-old male who presented with progressive skull swelling as the only symptom. Due to the lesion's pronounced mass effect and marked compression of the superior sagittal sinus, a craniotomy and complete surgical resection were performed. A systematic literature search was conducted via PubMed to identify previously reported cases of intracranial solitary plasmacytoma involving superior sagittal sinus compression.

**Results:**

In the present case, histopathological examination confirmed the diagnosis of solitary plasmacytoma and surgical resection alone resulted in a favorable clinical outcome without recurrence during a 40-month follow-up. Our systematic review identified 14 patients with intracranial solitary plasmacytoma causing superior sagittal sinus compression across 12 published articles.

**Discussion and conclusion:**

Differentiation from more common intracranial lesions is crucial and relies on careful radiologic and histopathologic evaluation. Solitary plasmacytoma should be considered in the differential diagnosis of dural-based lesions involving venous sinuses. Further studies and clinical reports are essential to clarify prognostic factors and to refine therapeutic strategies for this rare entity.

## Highlights

• Intracranial solitary plasmacytoma compressing the superior sagittal sinus is exceedingly rare.
• The lesion presented with features resembling common dural-based intracranial tumors.
• Total surgical resection led to excellent 40-month outcome without any recurrence.
• Literature review identified 14 similar cases with compression of the superior sagittal sinus.
• Radiologic and histopathologic features guiding differential diagnosis are detailed.

## 1 Introduction

Plasmacytoma is a hematological malignancy characterized by a solitary mass of neoplastic monoclonal plasma cells that can occur inside (solitary plasmacytoma of the bone, SPB) or outside (extramedullary plasmacytoma, EP) the bone (Kilciksiz et al., 2012). EP can present either as solitary tumors or as secondary manifestations of multiple myeloma (MM) (Dores et al., 2009). Solitary plasmacytoma represents a localized, typically benign neoplastic lesion of plasma cell origin, occurring in the absence of clinical, radiologic, or laboratory findings suggestive of systemic MM. However, it carries a risk of eventual progression to overt MM in a subset of cases (Schwartz et al., 2001). Thus, intracranial plasmacytoma or plasmacytoma of the skull have a wide spectrum of malignancy ranging from the quite benign solitary plasmacytoma and the malignant MM at its two ends (Dong et al., 2013). Notably, intracranial involvement resulting in neurologic deficits is exceedingly rare, with only sporadic cases documented in the literature to date. In the present report, we describe a patient who harbored a solitary intracranial plasmacytoma with no evidence of disseminated disease or systemic myeloma, where the significant mass effect rendered surgical resection necessary.

## 2 Case report

**Fig. 1:**
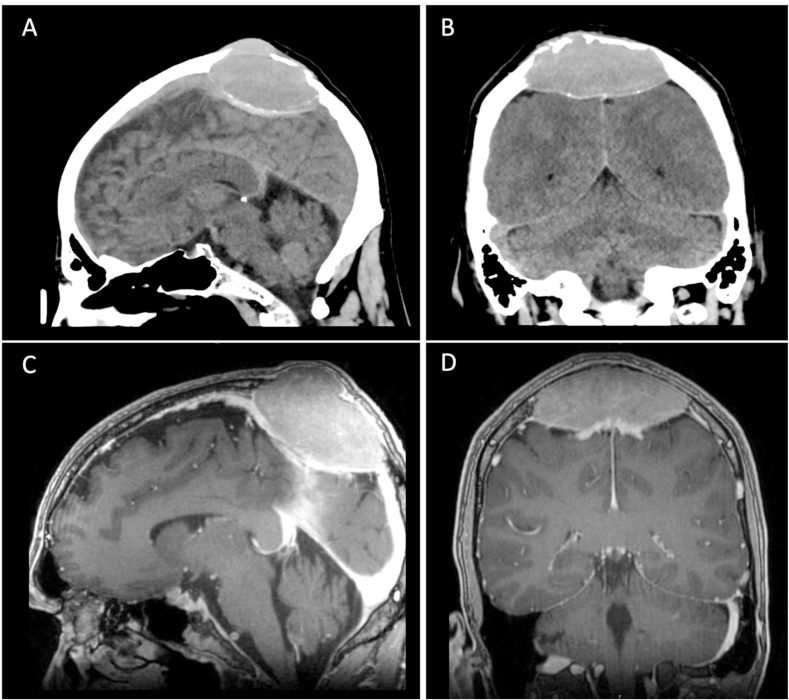
Preoperative imaging findings of the solitary intracranial plasmacytoma. A–B: Cranial CT (sagittal and coronal views) demonstrating a space-occupying lesion compressing the posterior third of the superior sagittal sinus (SSS), osteolysis of the skull, and expansion into the subcutaneous fatty tissue/into the cranial vault. C–D: T1-weighted MRI with contrast enhancement (CE-T1) (sagittal and coronal views) revealing homogeneous contrast uptake of the lesion, measuring 72 x 48 × 84 mm, suggestive of an osteomeningioma.

We present a 52-year-old male patient with a slowly progressive swelling of the skull, which the patient detected 10 months prior to medical consultation. At the time of initial evaluation by his general practitioner, the lesion was suspected to be a benign lipoma. An ultrasound was performed during the following examination nine months later and revealed a space-occupying lesion compressing the superior sagittal sinus (SSS) followed by further imaging with cranial computed tomography (cCT) and cranial magnetic resonance imaging (cMRI). Here, the tumor was found to compress the posterior third of the SSS with consecutive osteolysis of the skull (Fig. 1A and B). T1-weighted sequence with contrast-enhancement (CE-T1) showed a homogenous contrast uptake of the lesion with a dimension of 72 x 48 × 84 mm, highly suggestive of an osteomeningioma with osteolysis of the skull (Fig. 1C and D). T2-weighted sequences indicated no edema.

Neurological examination revealed no pathological findings. Preoperative blood sampling demonstrated a normal differential blood count and no evidence of systemic inflammation.

**Fig. 2:**
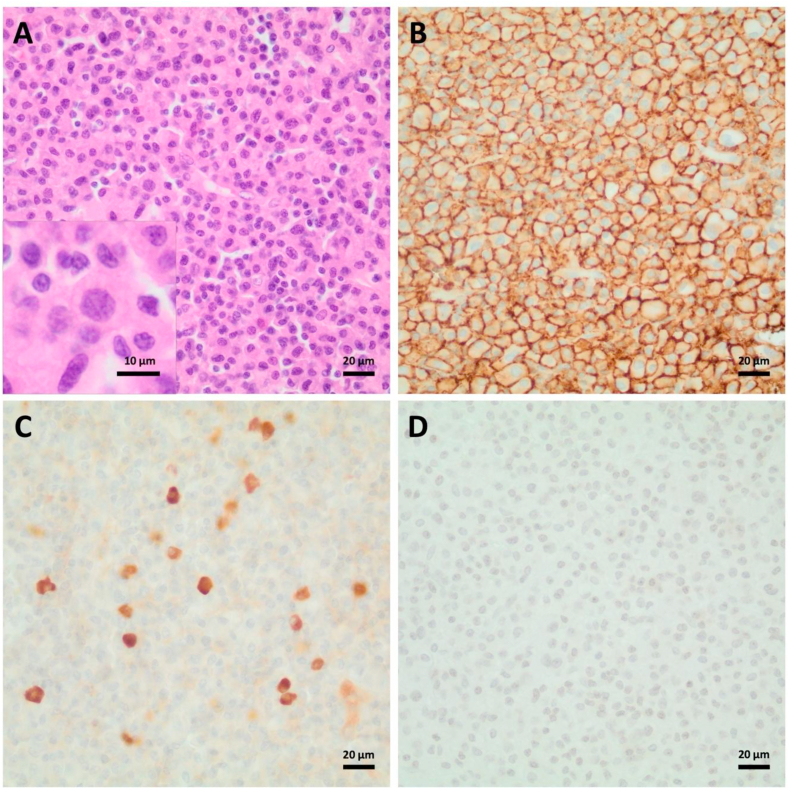
Histopthological examination revealed sheets of moderately pleomorphic neoplastic plasma cells with enlarged, slightly excentric nuclei with suggested clock-face chromatin pattern (A). The tumor cells showed strong positivity for CD138 (B) and occasional expression of kappa light chains (C), while being negative for lambda light chains (D).

Due to the large space-occupying lesion with compressive effect, the patient was admitted to our neurosurgical department for craniotomy and tumor resection, as preoperative imaging most likely indicated a meningioma exerting a mass effect. The surgical procedure was conducted under general anesthesia. An osteoplastic craniotomy was undertaken to provide adequate exposure of the lesion. Complete resection of the tumor was achieved, including excision of the involved segment of the dura mater to ensure clear margins and minimize the risk of residual disease. The tumor demonstrated a violet and livid discoloration with macroscopic hemorrhagic and necrotic segments. A duraplasty (DuraGen®) was laid on the resected parts. Polymethyl-methacrylate (PMMA) reconstructive cranioplasty was performed to repair the skull defect. Intraoperative examination of frozen sections indicated a preliminary differential diagnosis of either plasmacytoma or histiocytosis, necessitating further histopathological assessment for definitive classification. Postoperatively, the patient was monitored in our intensive care unit and developed no focal neurologic deficits. On the first postoperative day, the patient was transferred to our neurosurgical ward and the CT scan was unremarkable. The final neuropathological diagnosis was plasmacytoma with a Ki67-proliferation index of 5–10 % and expression of kappa light chain (Fig. 2).

**Fig. 3:**
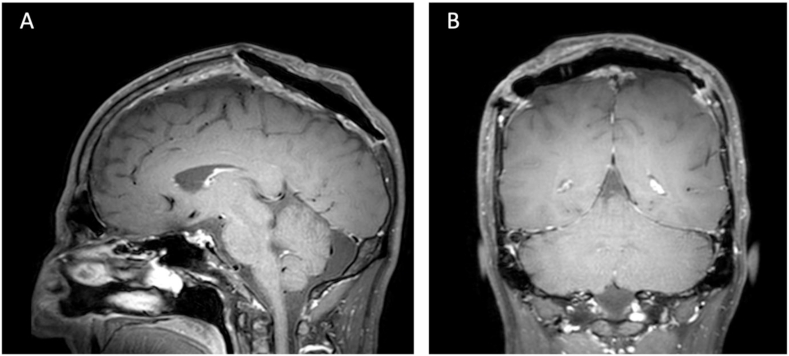
Postoperative MRI imaging following surgical resection. A-B: Postoperative MRI with contrast-enhanced T1-weighted black blood fat suppressed images (sagittal and coronal views) showing no evidence of residual tumor.

The patient was finally referred to the Department of Hematology and Oncology, among others for bone marrow puncture. Bone marrow histology revealed a normocellular marrow with normal trilineage hematopoiesis. Histologically and immunohistochemically, there was no signs of infiltration by MM. The patient had regular postoperative follow-up cMRIs (Fig. 3). He further did not underwent adjuvant radio- or chemotherapy postoperatively. Over a follow-up period of 40 months, there were no signs of recurrence or systemic progression detected through lab tests or imaging studies.

## 3 Literature review

This systematic review was conducted in accordance with the Preferred Reporting Items for Systematic Reviews and Meta-Analyses (PRISMA) guidelines to ensure methodological rigor and transparency. A targeted search strategy was developed to identify articles reporting on intracranial solitary plasmacytoma involving compression of the superior sagittal sinus, with PubMed selected as the primary database due to its extensive indexing of peer-reviewed literature. The search strategy utilized the terms “intracranial,” “brain,” “cerebral,” “sagittal sinus,” “solitary”, and “plasmacytoma” in various combinations to ensure the retrieval of relevant publications. Only articles describing solitary plasmacytomas were considered; cases involving multiple myeloma or systemic plasma cell disorders were excluded to focus exclusively on localized disease. Studies were included if they provided clear evidence of a solitary plasmacytoma with superior sagittal sinus compression, either explicitly described in the text or clearly identifiable on accompanying CT or MRI images. Publications were excluded if they were published prior to 1970, particularly when relying solely on outdated imaging modalities such as dynamic scintigraphy, due to insufficient anatomical detail. Additionally, reports lacking modern cross-sectional imaging (CT or MRI), or those in which sinus involvement could not be confidently verified, were also excluded.

**Fig. 4:**
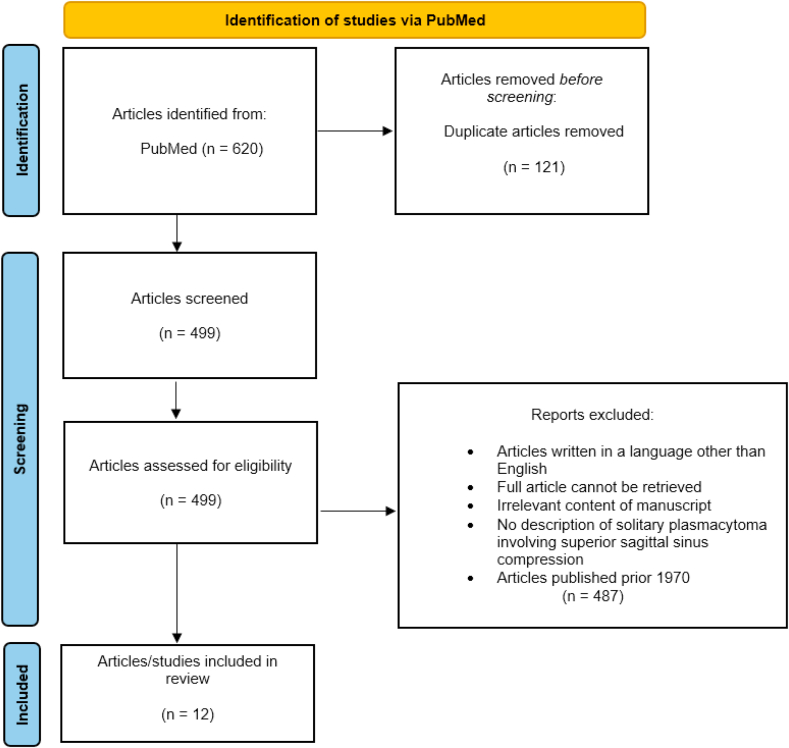
PRISMA flowchart.

The initial search yielded 620 articles, of which 121 duplicates were removed. The remaining 499 studies were screened by two independent reviewers (B.N.H. and M.S.-S.) based on titles and abstracts, applying the predefined inclusion and exclusion criteria. Following this screening process, 487 articles were excluded, resulting in 12 studies that met the eligibility criteria and were included in the final review. The flowchart of our literature search is presented in Fig. 4.

These 12 publications collectively reported on 14 patients diagnosed with intracranial solitary plasmacytoma causing superior sagittal sinus compression. The mean age across the pooled cohort was 52.7 years (range: 34–75 years), with an equal gender distribution (7 males and 7 females). The most commonly reported treatment approach was surgical resection followed by adjuvant radiotherapy. Among the studies that documented follow-up, the mean follow-up duration was 26.5 months. Table 1 summarizes the reported cases of solitary plasmacytoma with superior sagittal sinus compression.

**Table 1:** Summary of reported cases of intracranial solitary plasmacytoma involving compression of the superior sagittal sinus.

Authors	Year	Age (years)	Sex	Symptoms	Therapy	Outcome	FU
Harapan et al. *(our case)*	2025	52	M	Scalp swelling	Resection	No recurrence	40 months

Bouali et al. (Bouali et al., 2020)	2020	68	F	Headache, scalp swelling	Resection + postoperative radiotherapy (40 Gy)	No recurrence	28 months
Kuo et al. (Kuo et al., 2018)	2018	40	M	Scalp swelling	Preoperative embolization + resection	No recurrence	12 months
Khalili et al. (Khalili et al., 2015)	2015	47	M	Headache, hemiparesis, seizures	Resection	No recurrence	9 months
Devoe et al. (Devoe et al., 2014)	2014	75	F	Headaches, vertigo	Biopsy, radiation (2 × 45 Gy), lenalidomide	No recurrence	52 months
Azarpira et al. (Azarpira et al., 2014)	2012	34	M	Hallucination, amnesia	Resection + postoperative radiotherapy (50 Gy)	No recurrence	–
Cerase et al. (Cerase et al., 2008)	2008	67	F	Hemiparesis	Resection	No recurrence	45 months
Vaicys et al. (Vaicys et al., 1999)	1999	59	F	Seizures, hemiparesis, headaches	Resection, 5x intrathecal MTX (12 mg), radiotherapy (50 Gy)	No recurrence	6 months
Provenzale et al. (Provenzale et al., 1997)	1997	42 67	F F	Scalp swelling Hemianopsia	Biopsy, radiotherapy Subtotalresection + postoperative radiotherapy	– –	– –
Nagatomo et al. (Nagatomo et al., 1994)	1994	56	M	Scalp swelling	Resection + postoperative radiotherapy (40 Gy)	No recurrence	8 months
Kohli et al. (Kohli et al., 1982)	1982	49	F	Headache, nausea, personality changes	Resection + postoperative radiotherapy (40 Gy)	No recurrence	60 months
Jakubowski et al. (Jakubowski et al., 1980)	1980	47	M	Scalp swelling	Resection + postoperative radiotherapy	No recurrence	24 months
	1980	40	M	Headaches + scalp swelling	Resection + postoperative radiotherapy	No recurrence	12 months
Chang et al. (Chang et al., 1970)	1970	47	M	Headaches	Resection	No recurrence	36 months

## 4 Discussion

This rare case highlights the importance of recognizing solitary plasmacytoma as a potential diagnosis in common skull locations, despite its rarity. It underscores the diagnostic challenge of radiologic mimicry of meningiomas and the value of thorough differential diagnosis to avoid mismanagement. Moreover, it shows that complete surgical resection can provide durable control and emphasizes the essential role of multidisciplinary collaboration and individualized treatment planning.

Commonly, intracranial plasmacytoma is more often associated with MM than with solitary plasmacytoma. Unlike MM, solitary bone plasmacytomas are characterized by the absence of systemic involvement. Most cases follow a benign course, unless associated with MM. Although rare, cranial tumors may be the only manifestation of plasmacytomas, as observed in our case. Since some descriptive studies and reports do not clearly differentiate between solitary plasmacytomas and MM or considered plasmacytomas and MM in combination, disentangling potential differences in disease patterns is significantly hampered.

Solitary intracranial plasmacytomas frequently remain asymptomatic, however, depending on the location of the lesion, various symptoms might occur such as vision loss (Cao et al., 2010; Hogan et al., 2002; Chaar et al., 2003), ptosis (Bin Waqar, 2022), diplopia (Ibekwe et al., 2018; Kalwani et al., 2015; Kashyap et al., 2010; Bonduelle et al., 2021), eye pain (Matos et al., 2020), facial neuropathy (Mullaguri et al., 2018), photophobia (Soejbjerg et al., 2016), hypoglossal nerve palsy (Sin et al., 2015) or exophthalmos (Ashraf et al., 2003; Ahn et al., 2022). Secondary complications might arise from intracranial manifestations, e.g. cerebral venous thrombosis (with papilledema) (MacIntosh et al., 2017) and Gradenigo's syndrome (Bourne et al., 1998).

The role of imaging for solitary plasmacytoma is limited as imaging alone cannot determine these lesions with sufficient certainty since these lesions can mimic meningioma, diffuse leptomeningeal disease or lymphoma and thus complicate distinction (Ooi et al., 2006) as in our case. Furthermore, it seems that appearance on MRI scan may vary, a phenomenon described in another case report (Gregorio et al., 2019), where the extramedullary plasmacytoma – in contrast to our case – was surrounded by vasogenic edema.

Literature on the management of solitary cranial plasmacytoma is very limited due to their rarity. Although surgical resection of a solitary plasmacytoma may not be generally recommended, immediate debulking was indicated in our specific case to decompress adjacent structures, specifically the SSS and the motor cortex or central region.

A recent review has shown that for manifestations of extramedullary plasmacytoma in the soft tissues, surgical resection alone seems to be appropriate if the lesion is locally well-operable (Holler et al., 2022). In our case, gross total resection alone was also an effective treatment option.

A giant solitary plasmacytoma in a comparable location as in our patient has been previously described; however, in that case, embolization was performed prior to craniotomy, which marks a significant difference from our case (Kuo et al., 2018). Other intracranial locations of extramedullary plasmacytoma include the internal auditory canal and cerebellopontine angle (Lavinsky et al., 2016; Fujiwara et al., 1980), the posterior fossa (Daghighi et al., 2012; Rahmah et al., 2009; Weisberg, 1986), the skull base/orbital region (Wachter et al., 2010; Dang et al., 2019; Na'ara et al., 2016), falcotentorial location (Vaicys et al., 1999; Alappatt et al., 2004), the cavernous sinus (Tu et al., 2000), the sellar or para-/suprasellar region (Johnson et al., 2022; DiDomenico et al., 2018) and the cerebellum (Yanagihara et al., 2020). Lesions that affect the SSS seem to be very rare (Table 1), especially when the manifestation is a solitary plasmacytoma and not associated with MM.

Interestingly, intracranial solitary plasmacytoma or manifestations of MM can also present as subcortical intraparenchymal hemorrhage (Sato et al., 2022; Onodera et al., 2021) or subdural hematoma (Ozoner et al., 2018; Mitsos et al., 2004).

Our case report is notable for the following reasons: We demonstrate an unusual case of a solitary plasmacytoma of the skull and pronounced compression of the SSS, which was preoperatively classified as a meningioma and did not lead to edema in the cortical tissue despite the distinct compression of the SSS. Moreover, a retrospective analysis of 190 cases revealed that intracranial plasmacytomas most frequently occur along the calvarial convexity, particularly involving the frontal lobe and skull base (Feng et al., 2024), whereas lesions in the parietal region – as in our case – are considerably less common. We report a case of solitary intracranial plasmacytoma without evidence of systemic MM. The laboratory findings and bone marrow histology of our patient do not suggest dissemination and therefore point to a solitary extramedullary presentation intracranially, which is very uncommon.

In the case of a local space-occupying effect, the guideline therapy for SP consists primarily of radiation with a median dose of up to 45 Gy in addition to size reduction/resection and sample collection for diagnosis (Pham et al., 2019; Curry et al., 2020; Tsang et al., 2018). Various studies have shown that additional systemic therapy with, for example, lenalidomide has a possible positive effect on multiple myeloma-free survival (MMFS) and progression-free survival (PFS) (Mignot et al., 2020). This was also confirmed by the case reports we compiled on SP listed in Table 1, with an adjuvant radiotherapy in the vast majority. In our particular case, given the absence of any signs of local tumor recurrence and the findings of a normal bone marrow aspirate, adjuvant radiotherapy was deemed unnecessary and therefore not administered throughout the 40-month follow-up period.

To support diagnostic clarity, we present a detailed overview of the radiological (Table 2) and histopathological (Table 3) characteristics of intracranial solitary plasmacytoma and multiple myeloma, highlighting key aspects that aid in distinguishing these lesions from other dural-based or osteolytic cranial pathologies commonly encountered in neurosurgical differential diagnosis. According to a recent systematic review (Palavani et al., 2024), chordoma and meningioma represent the most frequently reported differential diagnoses in cases of intracranial plasmacytoma. Given the overlapping imaging and histopathological features, the differential diagnosis remains challenging, contributing to a high rate of preoperative misdiagnosis in patients with intracranial plasmacytoma (Feng et al., 2024).

**Table 2:** Radiological features of intracranial solitary plasmocytoma/multiple myeloma and their differential diagnoses.

Differential diagnoses	Common radiological features
**Solitary plasmacytoma/Multiple Myeloma**	Mostly purely lytic, sharply defined (“punched out”), possibly endosteal scalloping
**Meningioma**	Extra-axial location with dural tail, singular or multiple lesions, distinct contrast-enhancement, ± edema, ± diffusion restriction
**Lytic calvarial metastases**	Depending on the primary tumor
**Intraosseos epidermoid**	Very bright in DWI, scalloped border with a sclerotic rim
**Eosinophilic granuloma**	Sharp margins, beveled edges, diffuse contrast enhancement on MRI
**Intraosseous venous malformation (hemangioma)**	“Sunburst appearance”,“Wagon-wheel sign”or "honeycomb appereance "
**Paget disease (osteolytic phase)**	Usually preserved inner table in primary osteolytic stage
**Osteomyelitis**	Often located at the skull base
**Solitary fibrous tumor**	"yin-yang" appearance on T2, flow voids especially on surface, vivid heterogeneous contrast enhancement
**Cutaneus tumors with transcalvarian extension**	Depending on the primary tumor
**Bone tumors with transcalvian extension**	Depending on the primary tumor

**Table 3:** Histopathological features of intracranial solitary plasmocytoma/multiple myeloma and their differential diagnoses.

Differential diagnoses	Histopathological features
**Solitary plasmacytoma/Multiple Myeloma**	Sheets of plasma cells of varying degrees of differentiation, sometimes Russell bodies or amyloid deposition can be observed
**Meningioma**	Monomorphic cells arranged in lobules, whorls or fascicles, occasionally psammoma bodies
**Solitary fibrous tumor (SFT)**	Variable morphology with patternless or fascicular architecture with often abundant collagen deposition and thin-walled branching (staghorn) blood vessels
**MALT lymphoma of the dura**	Sheets of small mature B-lymphocytes of centrocyte-like or monocytoid appearance and plasmacytic differentiation
**Meningeal melanocytoma or melanoma (typically cervical and thoracic spine, rarely posterior fossa, very rare supratentorially)**	Nest-like, storiform, angiocentric or sheet-like arrangements of epitheloid or spindle-shaped tumor cells with variable amounts of melanin ranging from bland and well-differentiated (melanocytoma) to overtly malignant (melanoma)
**Epidural/subdural hematoma**	Desintegrated blood components (fresh hematomas); with increasing age accumulation of hemosiderophages and ferric iron, neovascularization and fibrohistiocytic organization
**Pituitary neuroendocrine tumor (PitNET; pituitary adenoma, if localized sellar or suprasellar)**	Well-differentiated monomorphic epitheloid tumor cells arranged in solid sheets, nests or perivascular pseudorosettes
**Langerhans cell histiocytosis**	Neoplastic Langerhans cells with enlarged grooved or reniform nuclei and abundant pale to eosinophilic cytoplasm admixed with reactive macrophages, lymphocytes, plasma cells and eosinophils, occasional multinucleated Touton giant cells may be observed
**Chordoma (when clivus/sphenoid bones are involved)**	Large clear-cell (physaliphorous) tumor cells arranged in chords and ribbons embedded in a myxoid matrix
**Chondrosarcoma (when clivus/sphenoid bones are involved)**	Chondrocytes embedded in lacunar spaces in an abundant cartilaginous matrix
**Metastatic carcinoma/Neoplastic meningitis**	Variably differentiated epithelial cells arranged in various growth patterns
**Schwannoma**	Biphasic growth pattern with compact Antoni A areas with often nuclear palisading (Verocay bodies) and loosely textured myxoid Antoni B areas

In summary, while solitary plasmacytoma as a disease entity is acknowledged in general terms, its intracranial manifestation with superior sagittal sinus compression remains undocumented in standard neurosurgical literature. This case contributes clinically relevant insights by presenting an uncommon topographic variant of solitary plasmacytoma involving the superior sagittal sinus, highlighting its radiologic resemblance to meningioma – a frequent diagnostic pitfall – and providing long-term follow-up data demonstrating excellent outcome after surgical resection alone. Furthermore, this report underscores the value of interdisciplinary tumor board discussions in determining the appropriateness of surgical monotherapy versus adjuvant treatment on a case-by-case basis.

## 5 Conclusions

Cranial plasmacytoma usually occurs as a manifestation of MM. Intracranial location is generally uncommon and should be considered in the differential diagnosis of lesions that radiographically mimic meningioma since management of these lesions might differ. Given that MM and solitary plasmacytoma share many histopathological features, yet differ significantly in terms of treatment protocols and prognostic outcomes, distinguishing between these entities is crucial. Solitary intracranial plasmacytoma is an extremely rare presentation in neurosurgical practice and is frequently misdiagnosed preoperatively, particularly since its imaging characteristics are nonspecific. The current body of knowledge on this condition is primarily derived from case reports. By contributing an additional well-documented case of solitary intracranial plasmacytoma with compression of the superior sagittal sinus, along with a focused systematic review of the literature, we aim to enhance the collective understanding of this rare presentation. Recognizing this entity in clinical practice may improve diagnostic accuracy and guide more effective, tailored therapeutic approaches – particularly where gross total resection may offer an effective treatment approach with the potential for favorable outcomes.

## Declaration of competing interest

The authors declare that they have no known competing financial interests or personal relationships that could have appeared to influence the work reported in this paper.
